# Phase Change Energy Storage Elastic Fiber: A Simple Route to Personal Thermal Management

**DOI:** 10.3390/polym14010053

**Published:** 2021-12-24

**Authors:** Weipei Li, Liqing Xu, Xiangqin Wang, Ruitian Zhu, Yurong Yan

**Affiliations:** 1 School of Materials Science and Engineering, South China University of Technology, Guangzhou 510640, China; liweipeidc@163.com (W.L.); xuliqingmm@163.com (L.X.); 2 Guangdong Medical Products Administration Key Laboratory for Quality Research and Evaluation of Medical Textile Products, Guangzhou Inspection Testing and Certification Group Co., Ltd., Guangzhou 511447, China; wangxq@gttc.net.cn

**Keywords:** porous structure fiber, hydrogen bond, elastic, phase change materials

## Abstract

A novel thermoplastic polyurethane (TPU) PCFs possessing a high loaded ratio and high elasticity was simply prepared by vacuum absorption following wet spinning, then coated by waterborne polyurethane (WPU). Octadecane (OCC), hexadecanol (HEO), and stearic acid (SA), which have different tendencies to form hydrogen bonds with TPU, were selected as PCMs, and their thermal behavior, thermal storge properties, and elasticity were systematically studied, respectively. The hierarchical pore structure though from the sheath to the core part of TPU filaments weakened the influence of the nonfreezing layer and hydrogen bond on the crystallization behavior of PCMs. The resulting HEO/TPU fiber has the highest enthalpy of 208.1 J/g compared with OCC and SA. Moreover, the HEO/TPU fiber has an elongation at break of 354.8% when the phase change enthalpy is as high as 177.8 J/g and the phase change enthalpy is still 174.5 J/g after fifty cycles. After ten tensile recovery cycles, the elastic recovery rate of HEO/TPU fiber was only 71.3%. When the HEO in the fiber was liquid state, the elastic recovery rate of HEO/TPU fiber promoted to 91.6%. This elastic PCFs have excellent thermal cycle stability, elastic recovery, and temperature sensitivity. It has great application potential in the fields of flexible wearable devices, intelligent fabrics, and temperature sensors.

## 1. Introduction

With the blooming of the population and the accelerated development of industrialization, the global energy demand has risen sharply [1]. In order to meet the heat demand, excessive burning of fossil energy such as coal, natural gas, or petroleum products has caused severe energy shortages and serious environmental pollution [2,3]. Additionally, there is a space and time difference between the supply and the demand of heat energy, resulting in the waste of heat energy [4]. Therefore, it is urgent to improve heat energy utilization and storage technology to achieve energy conservation and sustainable development [5,6].

Thermal energy storage (TES) technology effectively solves the intermittently and fluctuating problems of heat sources, making thermal energy management more flexible, efficient, and reliable [6,7]. It is a low-cost energy-saving technology with great potential. Commonly used TES can be classified into three categories, including sensible heat, latent heat, and reversible chemical heat storage [8]. Among them, the latent heat storage technology of phase change materials (PCMs) with high energy storage density, high phase change enthalpy, constant temperature regulation, and excellent thermal stability is considered to be the most effective energy storage technology [9]. PCMs involving significant latent heat absorbing and releasing at a constant transition temperature have been extensively utilized in building walls [10], intelligent fabrics [11], battery thermal management [12], infrared stealth technology [13], and other fields [14].

According to different phase transition behaviors, PCMs can be divided into four types: solid–solid, solid–liquid, solid–gas, and liquid–gas. Among them, solid–gas and liquid–gas PCMs release gas during the phase transition process, and the volume change is too large to be encapsulated. The latent heat of solid–solid PCMs comes from a change of crystal structure and fracture of hydrogen bond, so it is difficult to combine with other materials in practical applications [13,15]. Therefore, solid–liquid PCMs have become the most widely used TES materials because of their high latent heat density and small volume change. However, liquid leakage and solid rigidity of PCMs have remained a long-standing disadvantage in manufacture and utilization [16,17].

Phase change composites with stable shapes are always designed by embedding PCMs into porous supporting matrix [18], polymer molten blending or polymer solution blending [19], or polymeric microcapsules [20] to overcome the problems of leakage during repeated fusion and solidification of solid–liquid PCMs [21,22,23]. Other strategies are also developed by post-filling [24] or coaxial spinning [25] to obtain core-sheath conjugated fibers with PCMs to fulfill stable phase change fibers (PCFs) [11,26]. Li etc. [11] immersed polyethylene glycol (PEG) in porous graphene aerogel fibers and produced the PCFs with a loaded ratio of 80%, but its elongation at break was only 2.1%. Fabrics and wearable devices made of this fiber cannot provide good comfort to the wearer. Xia etc. [27] synthesized a novel PEG-derivatives@F-SiO_2_ as a form-stable PCM (FSPCM) to prepare melt-spun PA6-based PCFs. The elongation at break of PA6-based PCFs was 340%, but the enthalpy was only 11.1 J/g. Recently, Hu etc. [28] fabricated flexible photothermal PCFs by compositing PU and photothermal phase change microcapsules modified by polypyrrole (PPy). The micro-PCMs/PU composite fiber exhibited high enthalpy (184 J/g) and favorable elasticity (372% elongation), but the key point is that the tendency of microcapsules agglomeration is inevitable, which makes the preparation process complex, and the strength of fiber is only 0.54 MPa [9].

Because porous cavities of the support matrix provide a capillary force to prevent the leakage of liquid PCMs, the question of how to design a prospect carrier is the hotspot in research fields. Chen etc. [29] fabricated a carbon nanotube sponge-encapsulated paraffin wax composite by infiltrating paraffin into a porous carbon nanotube sponge. The interaction between the nanotube network and the paraffin wax results in enhanced phase change enthalpy and thermal conductivity compared to pure paraffin wax. So far, it has been reported that the interaction between porous carriers and PCMs affects the thermal storage capacity [30], phase change temperature [31], crystal structure [32], and undercooling [33]. Relative interactions include pore confinement [34], hydrogen bonding [35], and van der Waals force [29]. Kadoono etc. [36] studied the effect of pore size on the crystallization behavior of PCMs by encapsulating stearic acid (SA) in silicon-based SBA-15 and carbon-based CMK-3 porous carriers, respectively. The pore size of SBA-15 and CMK-3 was 7.6 and 3.5 nm, respectively. The crystallinity of SA in SBA-15 and CMK-3 was 35.6% and 30.8%, respectively, indicating that small pore size limits the crystallization behavior of PCMs. Feng etc. [37] encapsulated PEG in porous carbon and mesoporous silica, in which the pore size of porous carbon was smaller than that of mesoporous silica. The results proofed that the crystallinity of PEG in porous carbon was higher than that of mesoporous silica, because the hydrogen bonds formed between mesoporous silica and PEG hindered the molecular movement of PEG. Porous cavities can prevent the leakage of PCMs, but it will also hinder the crystallization behavior of PCMs. In order to solve this problem, we design a hierarchical pore structure and controlled the pore size distribution by changing the concentration of spinning dope. The pores on the sheath of fiber provided a capillary force to prevent the leakage of PCMs. The larger pores in the core part used as a storage cavity for PCMs crystallization. By changing the wet spinning process, the pore structure in the fiber was fabricated, and the influence of different pore structure on the PCMs was discussed.

In this paper, a novel high loaded ratio elastic TPU PCFs was fabricated by vacuum absorption PCMs into porous TPU fibers and coated by waterborne polyurethane (WPU). We chose octadecane (OCC), hexadecanol (HEO), and stearic acid (SA) as PCMs to study the effect of hydrogen bonds on the heat storage capacity of PCMs. The three kinds of PCFs with different phase change temperatures can be applied to different temperature environments. Moreover, TPU provided a good elasticity even at a high loaded ratio, and the solid–liquid transition of PCMs under the phase change temperature also changed the elastic recovery rate of the fiber. Thus, these PCFs have great application potential in the field of flexible wearable devices and temperature sensors. Woven fabric of the TPU PCFs kept constant temperature, can be used to adjust the thermal state of fabric, and can achieve an effect of intelligent thermal regulation.

## 2. Materials and Methods

### 2.1. Materials

N, N-Dimethylformamide (DMF, 99.5%) was purchased from Rich Joint., Shanghai, China. Stearic acid (SA, 99.5%) and octadecane (OCC, 99.5%) were purchased from Aladdin., Shanghai, China, and hexadecanol (HEO, 99.5%) from Macklin., Shanghai, China. Thermoplastic polyurethane (TPU, Elastollan 1185A) was purchased from BASF Co., Ltd., Ludwigshafen, Germany. Waterborne polyurethane (WPU, F0411) was provided from JiTian Chemical Co., Ltd., Shenzhen, China. All reagents were used without further purification. Deionized water was prepared in our laboratory.

### 2.2. Preparation of PCFs

**Figure 1:**
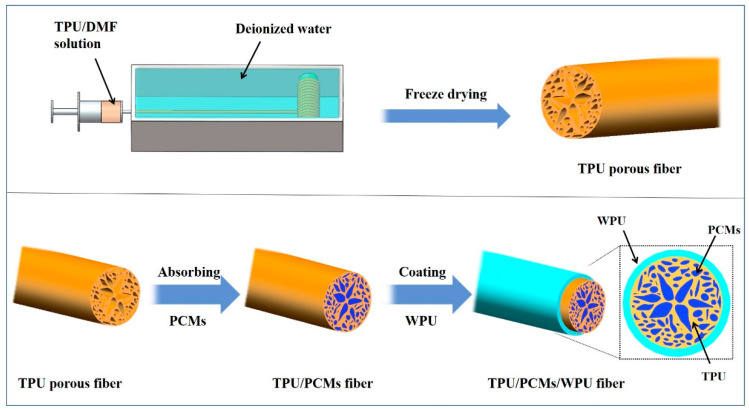
Flow chart for preparation of the elastic TPU PCFs.

A series of TPU PCFs with different loaded ratios were prepared by wet spinning, freeze-drying, vacuum absorbing, and coating technologies (Figure 1). The details were as follows: (1) a certain amount of TPU was dissolved in 25 mL DMF by agitation for 5 h at room temperature to obtain a uniform solution. The formulation of the elastic TPU scaffolds is shown in Table S1. (2) The spinning dope was extruded into a spin bath of deionized water through a flat-end needle (internal diameter 0.33 mm) at a rate of 50 μL/min. The as-spun fibers were placed in a spin bath for 30 min to stabilize its structure. (3) The as-spun fibers were freeze-dried (inside temperature −20 °C) to remove the residual solvent to prepare the porous structure. The pore fibers were named TPU-x (g/mL); x represents the concentration of TPU in the spinning solution, denoted as TPU-0.20, TPU-0.28, and TPU-0.40. (4) TPU-x was immersed in different molten PCMs in a vacuum environment for 3 h in order to fully penetrate PCMs into the pores. (5) A layer of WPU was coated on the surface of the fiber to ensure that the pores on the fiber surface were sealed. The PCFs were named Y/TPU-x, Y refers to the PCMs encapsulated in the fibers, denoted as OCC/TPU-x, HEO/TPU-x, and SA/TPU-x.

### 2.3. Characterization

The cross section and the surface morphology of the PCFs were recorded by scanning electron microscope (SEM, NOVA NANOSEM 430, FEI, Hillsboro, OR, USA). All samples were sprayed with gold.

The crystalline structure of PCMs in the fiber was analyzed by X-ray Diffraction (XRD) (X’Pert Pro, PANalytical, Almelo, The Netherlands). The test conditions were Cu kα target, tube voltage of 40 kV, tube current of 100 mA, scanning range (2θ) of 5~90°, and scanning rate of 4°/min.

The interaction between the TPU scaffold and PCMs was performed by Fourier Transform Infrared spectroscopy (FTIR) (MAGNA 760 model, Nicolet Instrument, MA, USA). The scanning range was 600~4000 cm^−1^ at room temperature, and the working mode was total reflection mode.

The thermal stability of PCFs was characterized by thermogravimetric analysis (TG 209 F1, Netzsch, Selb, Germany) at a heating rate of 10 °C under nitrogen atmosphere, and the test temperature range was 25 to 900 °C.

The performance of heat preservation of PCM/TPU fabric was characterized by an Infrared Thermal Camera (Vh-780, Infra Tec, Dresden, Germany).

The mechanical properties of PCFs were characterized by a single yarn strength tester (YG 021, Changzhou Second Textile Instrument Factory CO., Ltd., Zhejiang, China) according to ASTMD 3822 standard. The tensile speed of sample test is 25 mm/min and the clamping length is 5.0 cm. The elastic recovery rate was calculated according to the following equations [38]:

(1) $$\mathrm{Elastic}\;\mathrm{recovery}\;\mathrm{rate}=\frac{l_{\max}-l_{\mathrm{recovery}}}{l_{\max}-l_{0}}\;\times \;100\%$$

Among them, *l*_0_ is the initial length of fiber, cm; *l_max_* is a certain maximum length of fiber under stretching, cm; *l_recovery_* is the length of fiber after 10th extend-recovery cycles at room temperature, cm.

Hysteresis loss was calculated according to the following equations [39]:

(2) $$\mathrm{hysteresis}\;\mathrm{loss}=\frac{E_{0}-E_{r}}{E_{0}}\;\times \;100\%$$

Among them, *E*_0_ is loading work, J/m; *E*_r_ is unloading work, J/m.

The thermal storage properties of pure PCMs and composite PCFs were investigated using a differential scanning calorimeter (DSC) (204 F1, Netzsch, Selb, Germany). The scanning rate was 10 °C/min ranging from 20 to 85 °C under a nitrogen atmosphere. The retention time of both at 20 and 85 °C was set at 5 min during the measurement. The cooling curve and the second heating curve were used to analyze the thermal properties of the Y/TPU-X Fiber. Encapsulation efficiency (*E*, %) and heat storage capacity (*φ*, %) were calculated according to the following two equations [35]:

(3) $$E=\frac{\Delta H_{m,\mathrm{PCMs}/\mathrm{TPU}}+\Delta H_{c,\mathrm{PCMs}/\mathrm{TPU}}}{\Delta H_{m,\mathrm{PCMs}}+\Delta H_{c,\mathrm{PCMs}}}\;\times \;100\%$$

(4) $$\varphi =\frac{E}{R}\times 100\%$$

Among them, Δ*H_m,PCM/TPU_* and Δ*H_c,PCM/TPU_* are the melting enthalpy and crystallization enthalpy of the PCFs, J/g. Δ*H_m,PCMs_* and Δ*H_c,PCMs_* are melting enthalpy and crystallization enthalpy of pure PCMs, J/g. *R* is the mass fraction of PCMs in fibers.

The thermal performance of the Y/TPU-x was tested by DSC after fifty heating–cooling cycles. The enthalpy preservation rate (*η*, %) was calculated according to the following equation:

(5) $$\eta =\frac{\Delta H_{m,50}+\Delta H_{c,50}}{\Delta H_{m,1}+\Delta H_{c,1}}\times 100\%$$

Here, Δ*H_m,_*_1_ and Δ*H_c,_*_1_ are the melting enthalpy and crystallization enthalpy, respectively, J/g, measured before the circulation of the fiber. Δ*H_m,_*_50_ and Δ*H_c,_*_50_ are measured after the 50th cycles.

## 3. Results and Discussion

### 3.1. Structure and Morphology

**Figure 2:**
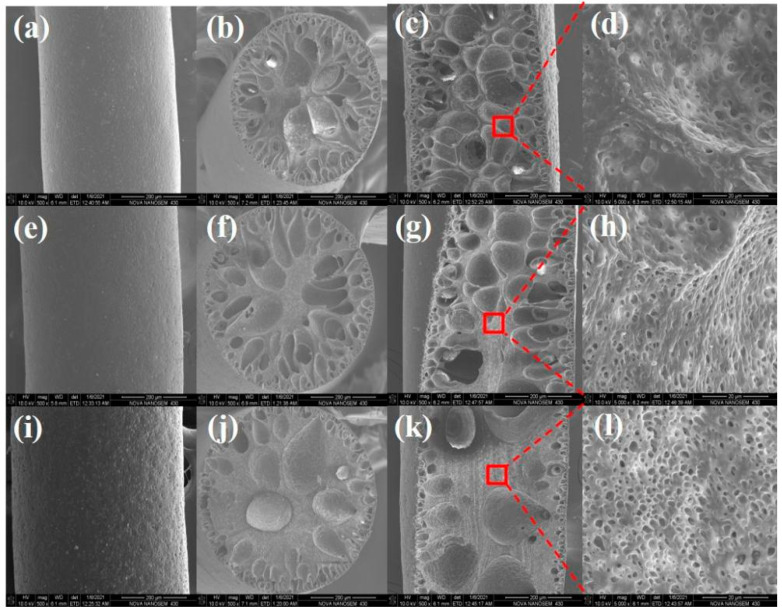
Influence of TPU spinning dope concentrations on structure inside the TPU fiber, the profiles of the surface (**a**,**e**,**i**), cross-section (**b**,**f**,**j**), and longitudinal section (**c**,**g**,**k**,**d**,**h**,**l**). and the dope concentration is 0.20 (**a**–**d**), 0.28 (**e**–**h**), and 0.40 g/mL (**i**–**l**), respectively.

As shown in Figure 2, many droplet-like macropore structures formed inside the TPU fibers. During wet spinning, the solvent exchange between the deionized water and the surface of TPU spinning dope took place rapidly, and the speed was related to the concentration of dopes. Because of the fast exchange of solvent and non-solvent between dopes and coagulation bath, the surface of the spinning dope immediately formed a dense structure, which prevented further exchange of the internal solvents inside the fiber with outside deionized water, and the process led to wrinkles and loosened structures inside the TPU fiber. Deionized water was located inside the loosened structures and provided the chance to form in situ droplet-like holes inside the TPU fiber with the help of a freeze-drying technique. The pores size and their distribution were the results of different solvent exchange rate and solidification rate between fiber surface and inner core part [40]. The larger pores were mostly located in the core part while the smaller and dense one dispersed in the sheath part of the fiber. An interesting result was that massive micro-scale pores with the size varying from 1~5 μm were found in the walls of pores (Figure 2d,h,l), which means pores did not completely separate from each other by walls, and the chance of the molten PCM to transmission between pores was becoming possible under a vacuum impregnation process. The porosity of the fiber became lower with the increase of TPU spinning dope concentration (Figure 2b,f,j), and the proportion of the micro-scale pore inside the TPU fiber increased obviously at the TPU dope concentration of 0.28 and 0.40 g/mL. Higher spinning dope concentration led to a slight increase of the diameter of porous TPU fiber at the same spinning process (Figure 2a,e,i) because the higher the concentration of spinning dope, the thicker.

The solidified sheath layer of fiber formed. The macroscopically distributed pore size inside the fiber is widely varied, covering the highest diameter of more than 50 μm and the smallest size of less than 5 μm. Small pores can provide a certain capillary force to stabilize the liquid PCM in the fiber [41], reducing the leakage possibility, while the large pores act as the storage cavity without affecting the crystallization behavior of PCMs. Taking into account nanoconfinement effect, plenty of pores, including both nano-scale pores and micro-scale ones, will affect the phase change enthalpy [42]. The distribution of different pore sizes on the cross-section of the porous TPU fibers prepared from different spinning dopes was shown in Table 1. We found that the area proportion of pores smaller than 5 μm increased from 6.83% to 46.86%, and the proportion of pores bigger than 50 μm decreased from 63.35% to 33.91% with the increase of the concentration of the TPU spinning dope.

### 3.2. Characterization of PCFs

#### 3.2.1. FT-IR

**Figure 3:**
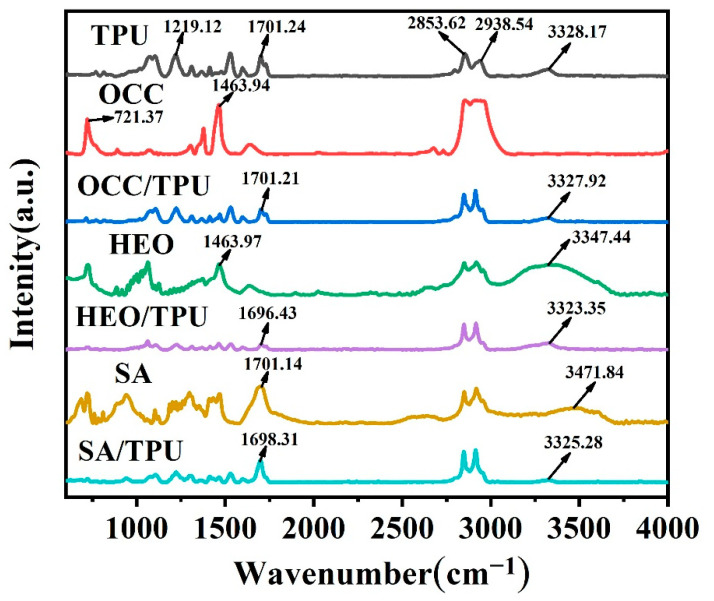
FT-IR spectra of TPU-0.28, PCMs, and PCM/TPU-0.28 fibers loaded different PCMs. Here, PCM was OCC, HEO, and SA, respectively.

TPU fiber prepared from the TPU spinning dope of 0.28 g/mL (TPU-0.28) was selected as a template fiber to package three PCMs, i.e., OCC, HEO, and SA, to study the interaction between TPU matrix and PCMs. According to Figure 3, the two strong peaks at 2938.54 and 2853.62 cm^−1^ were C-H stretching vibration peaks of TPU, the two peaks at 3328.17 and 1701.24 cm^−1^ were stretching vibration of N-H and carbonyl, respectively, and the peak at 1219.12 cm^−1^ corresponded to the irregular stretching vibration absorption peak of C-O-C [43]. In addition to the characteristic peak of C-H at 2800~3000 cm^−1^, the symmetric stretching absorption peak (1463.94 cm^−1^) and in-plane swing shock absorption peak (721.37 cm^−1^) of C-H belonged to OCC [44]. The characteristic peaks of HEO were the stretching vibration of O-H at 3347.44 cm^−1^, and SA of 1701.14 and 3471.84 cm^−1^ belonged to C=O and O-H, respectively. A special peak of HEO was at 3347.44 cm^−1^, which belonged to O-H. Since this peak was close to the characteristic peak of N-H, it was difficult to find the characteristic peak of O-H in HEO/TPU phase change fibers.

The stretching vibration peaks of the N-H and C=O groups of OCC/TPU fibers were at 3327.92 and 1701.21 cm^−1^, respectively, and the positions were similar to those of the pure TPU fiber. The N-H peaks of HEO/TPU and SA/TPU fibers were located at 3323.35 and 3325.28 cm^−1^, respectively, which were all red-shifted compared with the pure TPU fiber. At the same time, the characteristic peaks of C=O groups on the HEO/TPU and SA/TPU fibers were located at 1696.43 and 1698.31 cm^−1^, respectively, which also showed a red shift tendency compared with that of pure TPU fiber. Because of hydrogen bonds between the N-H in TPU and the hydroxyl group in HEO, or the carboxyl group in SA, shift of the absorption peak position of the N-H bond in the PCFs was found [45]. However, no hydrogen bond formed between OCC and TPU.

#### 3.2.2. XRD

**Figure 4:**
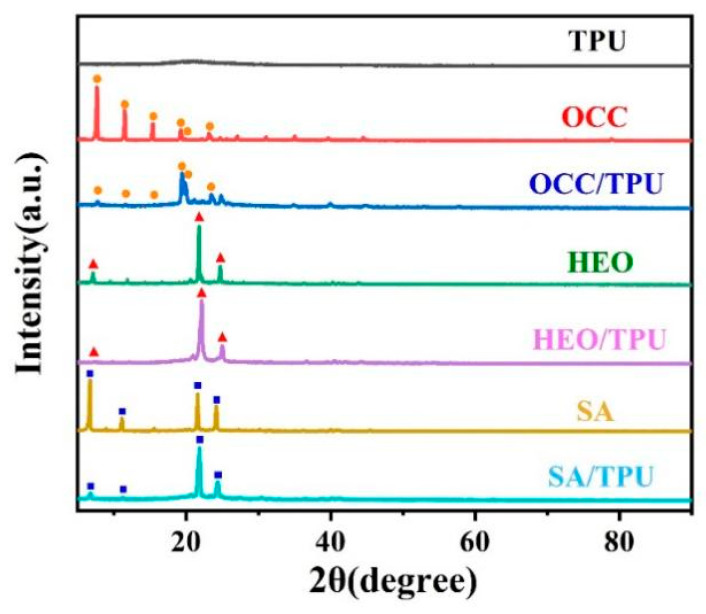
XRD diffraction of PCMs before and after encapsulation into TPU-0.28 fiber.

The crystallization behavior of PCMs is affected by the absorption of porous structure, its heat absorption and release ability are also affected by the melting and crystallization process [46]. Many pores formed inside TPU fibers, so it is necessary to explore the crystal structure of PCMs before and after encapsulation in TPU fibers. TPU does not crystallize at room temperature, and there is no obvious diffraction peak in XRD diffraction (Figure 4). The characteristic peaks in XRD diffraction belonged to the crystal forms of PCMs. For OCC, 2θ = 7.66°, 11.49°, and 15.41° diffraction peaks in XRD corresponded to (002), (003), and (004) diffraction crystal planes, which corresponded to β crystal form of OCC, 2θ = 19.28°, 19.84°, and 23.20° corresponded to (010), (011), and (105) diffraction crystal planes, which represented α crystal form of OCC [47]. Compared with XRD diffraction before and after OCC encapsulation, we found that OCC’s crystal was mostly in β form but changed to α form after encapsulated in TPU fiber. Because OCC was homogeneous nucleated before encapsulation, the pore surface provided many nuclei and promoted its heterogeneous nucleation after encapsulation, resulting in the difference of crystal forms. However, the intensity of characteristic peaks of HEO and SA slightly weakened after encapsulation, indicating that the confinement of pores changed the crystallization of HEO and SA. No new peaks in all curves were found, which means three PCMs did not form new substances inside the porous TPU fibers.

According to the Scherrer formula [48]:

*D* = *Kλ*/(*Bcosθ*) (6)

where *D* is the crystal size, *K* is the Scherrer constant, *λ* is the X-ray wavelength, *B* is the half-peak width of the characteristic diffraction peak, and *θ* is the Bragg diffraction angle. The crystal size of PCMs before and after encapsulation are listed in Table 2.

The crystal size of three PCMs were all reduced after encapsulation. Because the porous structure in the fiber provided abundant nucleation sites, the existent large number of crystal nuclei promoted the nucleation rate, but the growing of the crystals was limited, and the crystal size became smaller. For PCMs possessing high undercooling, the loaded matrix with high specific surface area provided more nucleation sites and reduced the undercooling [49].

### 3.3. Thermal Behaviors and Properties

#### 3.3.1. Thermal Storage Capability

**Figure 5:**
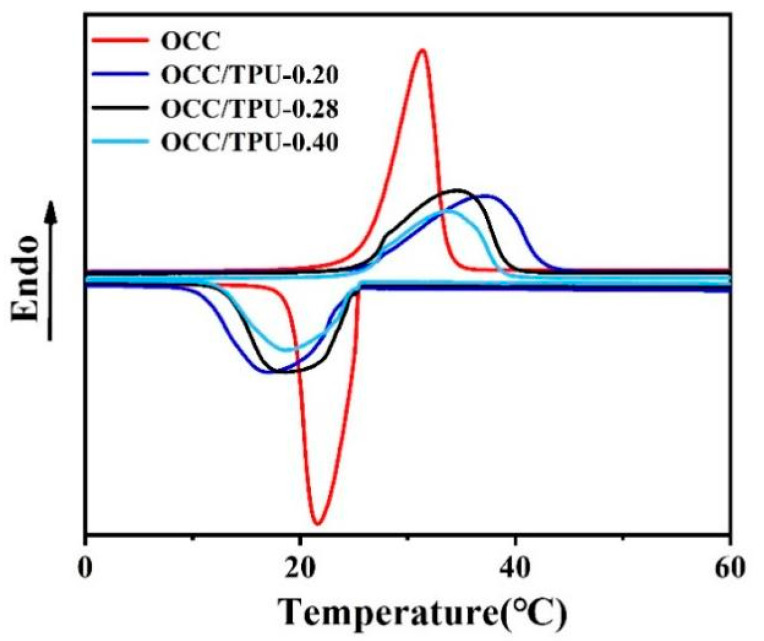
DSC curves of encapsulated OCC with different concentration spinning dopes.

In order to study the effect of pore size distribution inside TPU fiber on the heat storage capacity of PCMs, OCC was used, because no hydrogen-bond formed between OCC and TPU. From DSC curves (Figure 5), we found that the decrease of phase change enthalpy and encapsulation efficiency showed the same tendency with the fiber porosity decreases and the increase of TPU spinning dope concentration (Table 3). Porous TPU fibers do not contribute to the phase change enthalpy in the tested temperature range, so the phase change enthalpy of pure OCC is higher than the other three PCFs. On the other hand, the thermal conductivity of OCC encapsulated in the TPU scaffold is slower than that of pure OCC, so the peak shape becomes wider and shorter after being encapsulated [50]. Therefore, OCC/TPU PCFs have an adjustable phase change enthalpy, with the loaded ratio ranging from 56.2% to 76.9%, and the fusion enthalpy ranging from 127.7 to 176.1 J/g. In addition, we characterized the phase change behavior of fibers with different spinning dope concentrations and different PCMs, shown in Table S2.

Among them, *T_mo_* is the temperature at the beginning of melting; *T_mp_* is the temperature of melting peak; Δ*H_m_* is the melting enthalpy; *T_co_* is the temperature at the beginning of crystallization; *T_cp_* is the temperature of crystallization peak; Δ*H_c_* is the crystallization enthalpy; *E* is the encapsulation efficiency.

According to Table 4, we found that the percentage of the pore size less than 5 μm calculated from fiber section increased with the spinning dope concentration, the values were 6.83% for the spinning dope concentration of 0.20 g/mL, 13.44% for 0.28 g/mL, and 46.86% for 0.40 g/mL. *φ* is the heat storage capacity of three phase change materials encapsulated by fibers with the pore size distribution of 6.83%, 13.44%, and 46.86%.

Table 4 shows the relationship between the proportion of the pore size of less than 5 μm in PCM/TPU-x fibers and their heat storage capacities. It is found that as the proportion of small pores inside the TPU fiber increases, it promotes the tendency of PCMs not to undergo a phase change process [51], and the heat storage capacity decreases. On the other hand, big-size pores can be used as storage chambers for PCMs, which reduced this effect.

At the same time, it is found that the heat storage capacity of OCC/TPU fiber was slightly higher than that of HEO/TPU and SA/TPU fiber for all concentrations of spinning dopes. This phenomenon was the same in other studies. Qian etc. [52] encapsulated SA (-COOH), HEO (-CH_3_), and OCC (-OH) in mesoporous silica and found that OCC and SA formed hydrogen bonds with the surface of mesoporous silica, which restricted their movement, making the heat storage capacity of the composite only 27.4% and 52.7%, respectively.

#### 3.3.2. Thermal Durability

**Figure 6:**
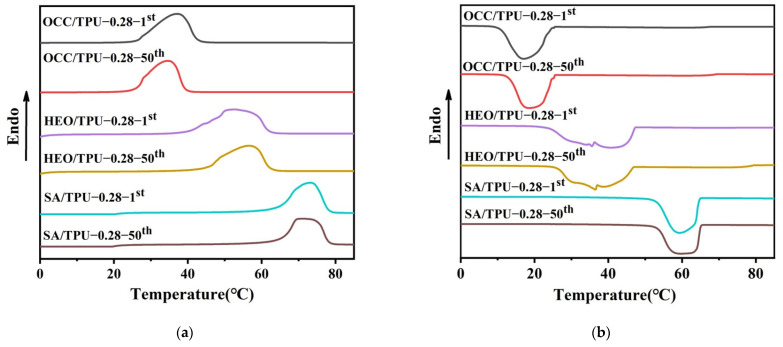
DSC curves of TPU-0.28 phase change energy storage before and after fifty thermal cycles: (**a**) heating curve and (**b**) cooling curve.

After fifty thermal cycles, the heating and cooling curves of Y/TPU-0.28 PCFs were basically unchanged (Figure 6). The melting enthalpy and crystallization enthalpy decreased slightly, which is the result of the thermal volatilization of the PCM during the heating and cooling process [53]. However, after fifty cycles, the enthalpy preservation rate of the three PCMs were still more than 94.3%, which indicates that the TPU PCFs possessed good thermal stability and repeatability (Table 5). Besides, the thermal stabilities of PCMs, TPU-0.28 and Y/TPU-0.28 fibers were characterized by TGA and results are in Figure S1 and Table S3.

#### 3.3.3. Encapsulation Stability

**Figure 7:**
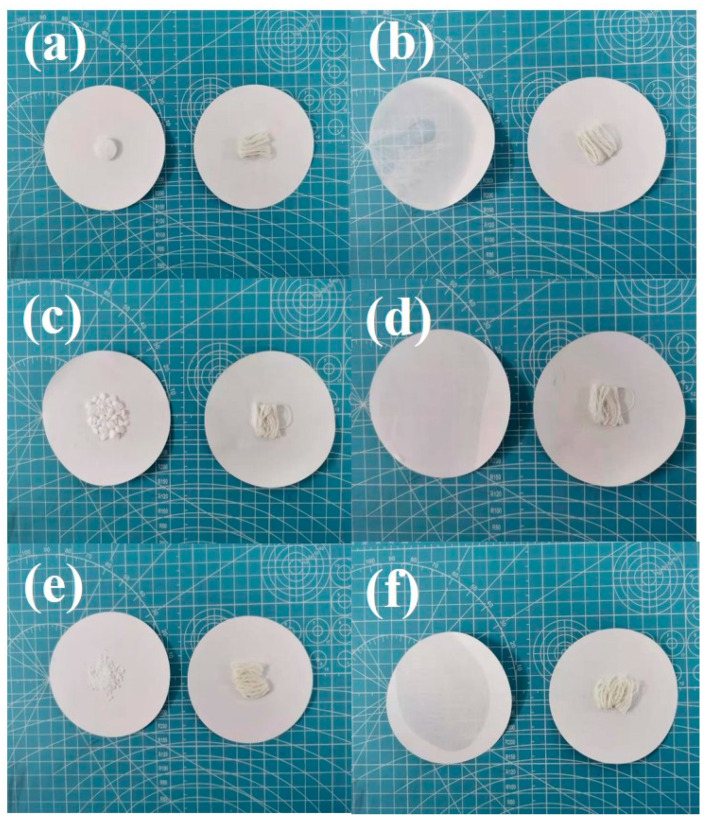
Digital photo of PCMs and Y/TPU-0.28 fibers before and after being heated: OCC: (**a**) before and (**b**) after; HEO: (**c**) before and (**d**) after; SA: (**e**) before and (**f**) after.

In order to characterize the encapsulation stability, PCMs and PCFs were heated in an oven for 1 h. When PCMs were heated higher than its phase change temperature, it changed from solid to liquid and penetrated into the filter paper, while the morphology of PCFs remained unchanged. After treated at 85 °C for 1 h, PCMs in the PCFs did not leak on the filter paper (Figure 7), because the pore structure inside the TPU fiber provided capillary force to fix the PCMs molecules and prevented their leakage. Moreover, the PCMs were further packaged in the porous TPU matrix by the surface-coated WPU.

#### 3.3.4. Thermal Insulation Performance of Fabric

**Figure 8:**
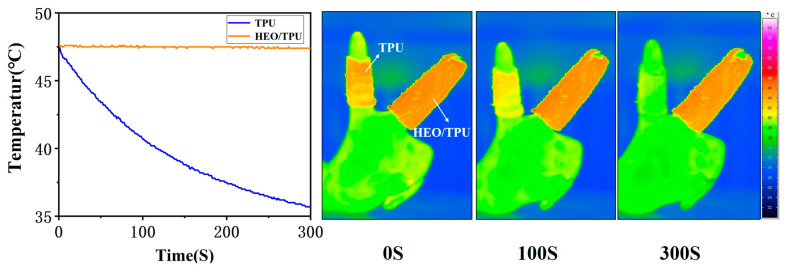
Infrared image of pure TPU-0.28 and HEO/TPU-0.28 fabric during cooling: pure TPU fabric on the left and HEO/TPU fabric on the right.

Pure TPU and HEO/TPU-0.28 fibers were woven into fabrics, respectively. The diameter of a pure TPU fabric sleeve is 2.5 cm, and the length is 4.5 cm. The diameter of HEO/TPU-0.28 fabric sleeve is 3.0 cm, and the length is 7.5 cm. The two fabrics were heated in an oven and then cooled down at room temperature. The cooling process was recorded with an infrared image, as shown in Figure 8. It is found that, at the same ambient temperature, the pure TPU fabric quickly dropped to room temperature, while the surface of HEO/TPU-0.28 fabric remained at 45 °C. In order to better verify the thermal insulation performance of composite phase change fabric, a traditional cotton fabric, TPU-0.28 fabric, OCC/TPU-0.28 woven fabric, and HEO/TPU-0.28 woven fabric were heated and maintained at 65 °C and then cooled down to room temperature at ambient temperature. 

**Figure 9:**
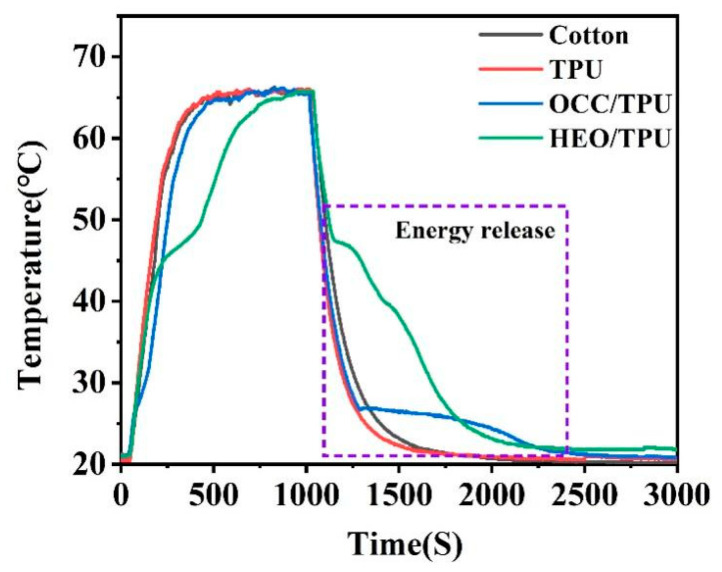
Temperature–time curves of fabrics made of cotton, TPU-0.28, OCC/TPU-0.28, and HEO/TPU-0.28 under heating and cooling.

These fabrics was prepared by winding the fibers on a glass slide in a rectangular shape with a size of 2.5 cm × 1.0 cm × 0.3 cm. The temperature change curve was shown in Figure 9. Interesting results show that cotton fabric and TPU fabric had the same heating–cooling trend, while OCC/TPU-0.28 woven fabric and HEO/TPU-0.28 woven fabric showed a temperature platform during the heating stage, which covers the molten temperature of PCMs. The platform is the results of the absorbed heat and melt of PCMs inside the fiber, which makes the fabric temperature maintain a stable temperature. When PCMs are completely molten, the fabric temperature began to rise again. 

The temperature plateau of HEO/TPU-0.28 was wider than that of OCC/TPU-0.28 because the temperature difference between the fabric and the environment shown a great influence on the heat absorption tendency of the fabric. The phase transition temperature of OCC is 28 °C, and HEO is 46 °C. The endothermic temperature difference of OCC is 37 °C, and HEO is 19 °C. Therefore, OCC has a higher heat absorption power than HEO and undergoes complete phase transformation in a short time. In the cooling stage, the cotton and TPU-0.28 fabrics cooled down to room temperature immediately, while OCC/TPU-0.28 woven fabric and HEO/TPU-0.28 woven fabric still showed a temperature plateau, respectively. PCMs inside PCFs fulfilled the crystallization and released heat, which was stored during the heating process. After encapsulating PCMs, the fabric can store latent heat and change the temperature buffer when the temperature changes dramatically.

### 3.4. Thermal Behaviors and Properties

**Figure 10:**
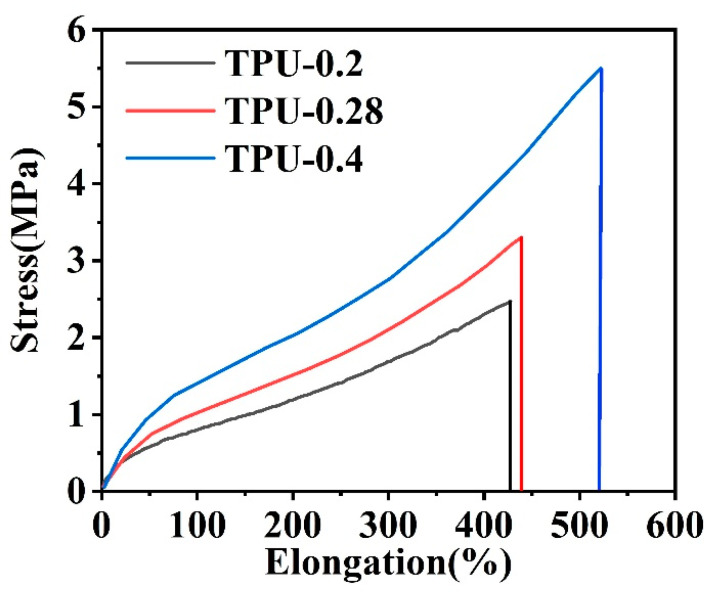
Tensile curves of porous TPU fibers prepared with different spinning dope concentrations.

The tensile curves of porous TPU fibers prepared with different spinning dope concentrations are shown in Figure 10. It is found that the tensile strength and the elongation at break of the porous TPU fiber increased with the polymer spinning dope concentration. Considering SEM analysis, the higher the concentration of the spinning dope, the easier it is for the fiber to form a dense structure, which is directly related to the mechanical properties of the fiber.

**Figure 11:**
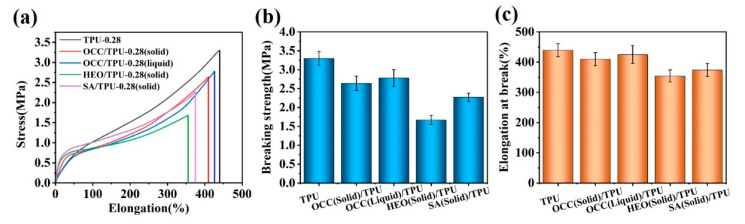
Mechanical properties of TPU-0.28 and PCM/TPU-0.28 fibers: (**a**) tensile curves; (**b**) tensile strength; and (**c**) elongation at break.

Figure 11a shows the tensile curves of TPU-0.28 fiber and Y/TPU-0.28 fibers. All stress–strain curves showed a classical curve of high elastic polymer [54], indicating that the main stress-loading medium of the PCFs was the porous TPU fiber. However, the curve of Y/TPU-0.28 fibers showed higher initial slops than that of TPU-0.28, which means higher modulus of PCFs were the results of PCMs encapsulated inside the porous TPU fiber. The solid PCMs have a certain mechanical strength, and act as a reinforcing component under small stress. For molten OCC/TPU-0.28 fiber, it is found that its initial modulus was similar to that of TPU-0.28 fiber, because the liquid OCC had almost no strength and could not influence the mechanical properties of OCC/TPU-0.28 fiber. However, the fracture strength and elongation at break of Y/TPU-0.28 fiber were lower than those of pure TPU fiber under high stress (Figure 11b,c). Because the fiber shrinks when entering the second stage of stretching, the solid PCMs in the pores hindered the shrinkage tendency, so that the porous TPU fibers were subjected to longitudinal and transverse forces at the same time [26]. Although the mechanical properties of PCFs are lower than those of TPU fibers, they still showed reliable mechanical properties.

The OCC/TPU-0.28 and SA/TPU-0.28 fibers prepared via wet spinning and vacuum impregnation achieved a higher loaded ratio and higher elasticity compared to previously reported PCFs prepared by different approaches, and their thermal properties and elastic properties were summarized in Table 6. In addition, we also discussed the effect of spinning dope concentration on the mechanical properties of the fiber, shown in Table S4. As the TPU fiber becomes denser, the tensile strength and the elongation at break of it increase to varying degrees.

**Figure 12:**
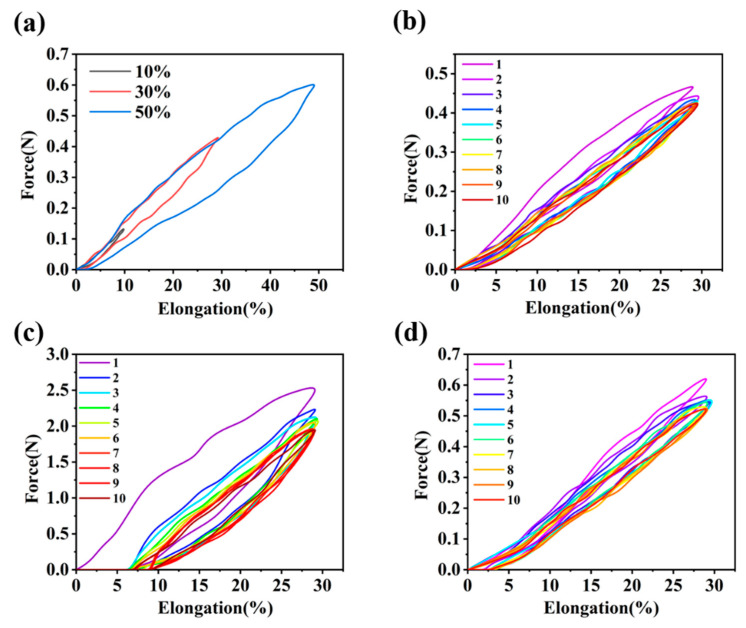
Resilience curves of TPU-0.28 fiber and HEO/TPU-0.28 fiber: (**a**) resilience performance of TPU-0.28 fiber under different elongation; (**b**) resilience performance of TPU-0.28 fiber after ten cycles at 30% elongation; (**c**) resilience curves of solid HEO/TPU-0.28 fiber after ten cycles; (**d**) resilience curves of liquid HEO/TPU-0.28 fiber after ten cycles.

Figure 12a shows the resilience performance of TPU-0.28 fiber at different elongations. As the elongation increases, the tensile stress of the fiber also rose up. During the rebound process, the recovery rate of the elastic TPU-0.28 fibers under the 50% elongation was lower than that of under the 10% elongation, because the visco-elastic nature of elastic polymer and the higher tensile stress leads to the stress-relaxation [62]. The greater the fiber deformation was, the more difficult it was to restore to its original size, so the force measured during the rebound process of the high-elongation fiber illustrated a value less than that of the fiber undergoing low-elongation [63]. Even at 50% elongation, the TPU-0.28 fiber still possessed a good elastic recovery ability. Figure 12b shows the resilience performance curves of TPU-0.28 fiber after ten cycles of 30% elongation. We found that the force to extend the fiber to 30% elongation at the first cycle was higher than those of the other nine cycles. Repeated stretching and recovering in a short period of time made the fiber difficult to return to its original state completely, and the required force decreased. However, the porous TPU-0.28 fiber still had good elastic recovery performance after ten cycles. The resilience performance of HEO/TPU-0.28 fiber during ten cycles was shown in Figure 12, and the HEO was in solid state (c) and in molten state (d), respectively.

Similar to TPU-0.28 fiber, the required force for 30% elongation of HEO/TPU-0.28 fiber in the first cycle was higher than those of the other nine cycles, and the difference was more evident. The first stretching process caused the solid HEO inside HEO/TPU-0.28 fiber to deform and break, which hindered the movement of the elastic TPU matrix at the rebounding stage, so the fiber cannot fully recover to its original state at 30% elongation.

After ten tensile recovery cycles, the elastic recovery rate of HEO/TPU-0.28 fiber was only 71.3%. When HEO was in liquid state, the elastic recovery rate of the fiber promoted to 91.6% after ten cycles, as shown in Figure 12d. The resilience curve of HEO/TPU-0.28 fiber showed the same tendency to the TPU-0.28 fiber, but the force for the same elongation was improved.

**Figure 13:**
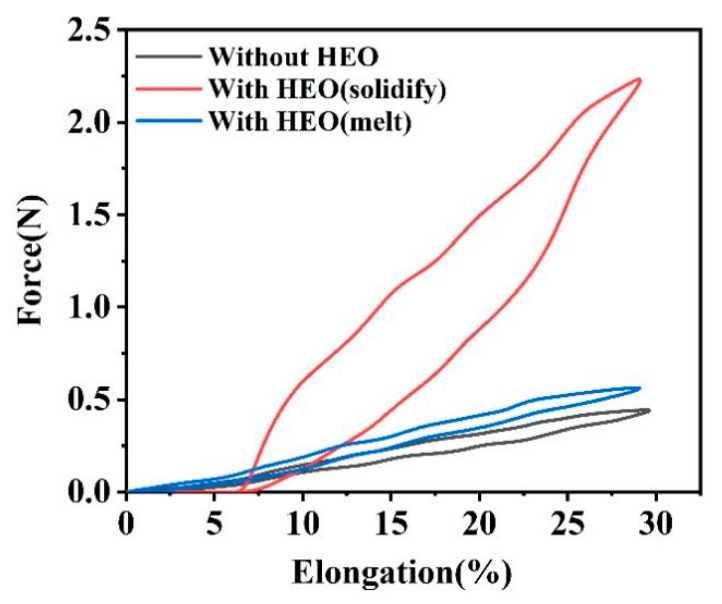
The second cycle resilience curve of different elastic fibers at 30% elongation.

We found that the required force to 30% elongation of HEO/TPU-0.28 fiber was higher than that of TPU-0.28, which means HEO improved the mechanical property of TPU. Taking the second cycle elasticity performance curve, for example, curves of TPU-0.28, HEO (solid)/TPU-0.28, and HEO (liquid)/TPU-0.28 fiber at 30% elongation were shown in Figure 13. The hysteresis loss of multi-hysteresis curves of Figure 12 and Figure 13 are shown on Table 7. It is found that the loading work, unloading work, and hysteresis loss of solid HEO/TPU-0.28 fiber are much higher than those of pure TPU fiber and liquid HEO/TPU-0.28 fiber, while pure TPU fiber and liquid HEO/TPU-0.28 fiber are almost the same. The state of PCMs in PCFs affected the elasticity of the fiber, so the elastic response of PCFs can be controlled by adjusting the temperature, which can be applied to the field of temperature sensors. After encapsulated HEO, the mechanical properties of HEO/TPU-0.28 fiber slightly improved in liquid state, and it still has a good elastic recovery ability. The solid PCMs have a supporting effect, which provided the fiber with a higher modulus. At the same time, it hindered the rebound of the porous TPU fiber, so that the fiber cannot return to its original size. This means if the fiber is heated to the phase change temperature, the influence of the solid PCMs on the resilience of TPU fiber can be eliminated. Moreover, it can return to its original size.

**Figure 14:**
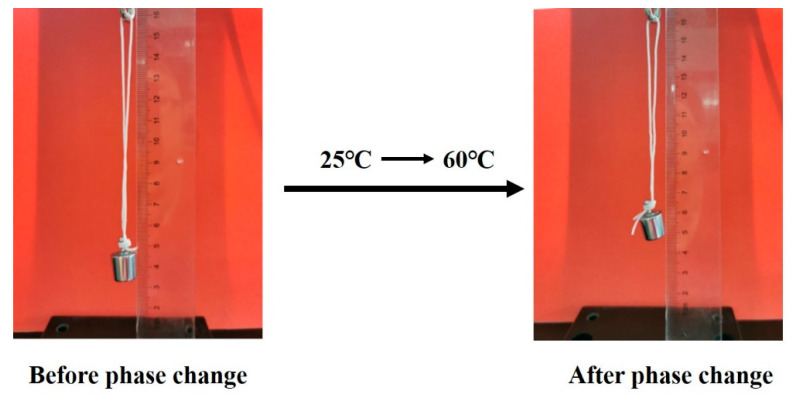
Changes of pre-stretched HEO/TPU fiber before and after heating.

As shown in Figure 14, the temperature sensitivity of HEO/TPU fiber was explored by pre-stretching. The fiber naturally shrinks after being stretched to 150% of its original length at room temperature, and then a weight (10 g) is bound at the bottom of the fiber to heat the fiber above the phase transition temperature and observe the recovery of fiber length. The weight (10 g) was suspended under the fiber, and the scale at the bottom of the weight recorded at 3.4 cm. After heating the fiber to the phase transition temperature of HEO, its length reduced, and the scale on the bottom of the weight was 4.8 cm, indicating that the fiber shortened by 1.4 cm. Solid–liquid mutual conversion of PCMs often occurs in PCFs, so even if the resilience of the fiber decreases in the solid state, it can return to its original size after the phase change behavior occurs. This kind of temperature-induced strain may also be applied to the field of thermal inductors.

## 4. Conclusions

In this study, we fabricated elastic porous TPU fibers by wet spinning and freeze-drying methods; then, different types of PCMs were encapsulated in the fibers by a vacuum impregnation and coated by a layer of WPU to prepare elastic PCFs. Differential scanning calorimetry was used to study the thermal storage performance of the PCFs. The highest loaded ratios of OCC/TPU, HEO/TPU, and SA/TPU fibers were 76.9%, 79.6%, and 84.2%, respectively. The highest phase change enthalpies were 176.1, 208.1, and 174 J/g, respectively, and the heat storage capacity was above 94.8%. The loaded ratio and heat storage capacity of these PCFs are higher than most reported PCFs, which is of guiding significance for the manufacture of PCFs with high heat storage capacity. By adjusting the pore structure of the TPU fiber, it is found that the pore confinement effect and hydrogen bonds limit the phase change behavior of PCMs, which reduces the heat storage capacity of it, and the hierarchical pore structure of TPU can alleviate these effects. When the proportion of pores with a diameter of less than 5 μm increases from 6.83% to 46.86%, the heat storage capacity of the PCFs just reduced by 2~2.5% according to different PCMs. The results show that even if the proportion of pores with a diameter of less than 5 μm is increased, the heat storage capacity of PCFs is still high, indicating that the hierarchical pore structure has great advantages. The thermogravimetric test and thermal cycle test show the fiber’s good thermal stability and durability. The phase change enthalpy of the HEO/TPU fiber was 177.8 J/g, and it was still 174.5 J/g after fifty thermal cycles. HEO/TPU fiber displayed excellent mechanical properties, such as high elongation at break of 354.8%. When the HEO in the fiber was liquid, the elastic recovery rate of HEO/TPU fiber was still 91.6% after being cyclically stretched for ten times at 30% elongation. The elastic HEO/TPU fiber has excellent thermal cycle stability, flexibility, and elastic recovery, and has the potential to apply in flexible wearable electronic devices and intelligent fabrics. The hysteresis loss of liquid HEO/TPU fiber and pure TPU fiber is basically the same, while the hysteresis loss of solid HEO/TPU fiber is much higher than that of liquid HEO/TPU fiber and pure TPU fiber, indicating that the state of HEO in HEO/TPU PCFs affected the resilience of fiber. The pre-stretching test of HEO/TPU fiber shows that, after heating to the phase transition temperature, the fiber recovered to its original size, indicating that the fiber has temperature sensitivity and has potential applications in the field of temperature sensors and intelligent fabrics. Compared with the OCC/TPU composite fiber without hydrogen bonds, the heat storage capacity of HEO/TPU and SA/TPU fiber was reduced by 1.5 to 3%, indicating that the hydrogen bond formed between HEO, SA and TPU macromolecular chain reduced the heat storage capacity of PCMs. The mechanical performance test shows that the tensile strength of the OCC/TPU fiber reached 4.18 MPa, and the elongation at break was 478.3%. To sum up, we prepared an elastic PCF, which has high elastic recovery rate and flexibility with a high loaded ratio. The elastic PCF also has temperature sensitivity and can potentially be used in the fields of flexible wearable devices, intelligent fabrics and temperature sensors.

## Figures and Tables

**Table 1:** The area distribution of the different size pores inside porous TPU fiber cross-section.

Fiber	<5 μm/%	5~50 μm/%	>50 μm/%
TPU-0.20	6.83	29.82	63.35
TPU-0.28	13.44	31.83	54.73
TPU-0.40	46.86	19.23	33.91

**Table 2:** Crystal size of PCMs before and after encapsulated in PCM/TPU-0.28 fibers.

PCMs	Before/nm	After/nm
OCC	39.11	25.15
HEO	46.89	19.32
SA	41.46	24.23

**Table 3:** Phase change behavior of OCC/TPU-X fiber.

Sample	*T_mo_* (°C)	*T_mp_* (°C)	Δ*H_m_* (J/g)	*T_co_* (°C)	*T_cp_* (°C)	Δ*H_c_* (J/g)	*E* (%)
OCC	26.4	31.4	226.0	25.4	21.6	230.1	-
OCC/TPU-0.20	25.8	37.2	176.1	24.1	17.1	174.6	76.9
OCC/TPU-0.28	26.4	35.0	154.2	25.0	17.9	157.8	68.4
OCC/TPU-0.40	26.3	33.7	127.7	24.8	18.7	128.8	56.2

**Table 4:** The relationship between the proportion of the pore size less 5 μm in Y/TPU-x fibers and their heat storage capacity (*φ*).

PCMs	*φ*/%		
	6.83/TPU-0.20	13.44/TPU-0.28	46.86/TPU-0.40
OCC	99.83	98.08	97.83
HEO	97.24	96.37	94.81
SA	98.75	96.97	96.64

Y/TPU-x here, Y means the percentage of pore size less 5 μm in fiber section area, and x is the spinning dope concentration of TPU solution.

**Table 5:** Phase change behavior of Y/TPU-0.28 PCFs before and after 50 cycles.

Sample	Number of Thermal Cycles	Δ*H_m_* (J/g)	Δ*H_c_* (J/g)	*η* (%)
OCC/TPU-0.28	1	154.2	157.8	96.7
	50	150.2	151.6	
HEO/TPU-0.28	1	177.8	185.1	94.3
	50	174.5	182.3	
SA/TPU-0.28	1	157.1	161.4	98.3
	50	150.3	157.9	

**Table 6:** Comparison of thermal and mechanical properties of PCFs prepared by different encapsulated approaches.

PCMs	Matrix	Strategy for PCMs Loading	Δ*H_m_* (J/g)	*E* (%)	Breaking Elongation (%)	Reference
Paraffin	Cellulose	Microcapsule and wet spun	69.01	48.06	2.0	[55]
Paraffin	Cellulose	Microcapsule and wet spun	130.34	77.28	1.2	[55]
Octadecane	Polypropylene	microcapsule and melt spun	11.00	20.00	30.2	[56]
Octadecane	Polyacrylonitrile-vinylidene chloride	Microcapsule and wet spun	30.00	30.00	7.0	[57]
Octadecane	Polypropylene	Microcapsule and melt spun	9.20	12.00	-	[58]
Paraffin	Polyvinyl alcohol	Microcapsule and wet spun	23.70	25.10	14.9	[59]
Paraffin	Polyvinyl butyral	Coaxial spun	128.20	50.00	110	[60]
Mixture of plant oils	Polyvinyl alcohol	Electro spin	84.72	70.00	83.77	[61]
Polyethylene glycol	Graphene aerogel fiber	Porous impregnation	188.40	84.20	2.5	[11]
Octadecane	Thermoplastic polyurethane fiber	Porous impregnation	176.10	76.90	364	This study
Stearic acid	Polyurethane fiber	Porous impregnation	174.00	84.20	253.4	This study
Stearic acid	Polyurethane fiber	Porous impregnation	148.30	72.00	498	This study

**Table 7:** Hysteresis losses of multi-hysteresis curves in Figure 12 and Figure 13.

Sample	Loading/(J/m)	Unloading/(J/m)	Hysteresis Loop/(J/m)	Hysteresis Loop/%
Elongation 10% TPU-0.28	0.619	0.552	0.066	15.763
Elongation 30% TPU-0.28	6.820	5.511	1.30	19.194
Elongation 50% TPU-0.28	17.535	12.033	5.502	31.377
Elongation 30% Liquid HEO/TPU-0.28	9.035	7.500	1.535	16.989
Elongation 30% Solid HEO/TPU-0.28	30.886	20.500	10.386	33.627

## Data Availability

Data are contained within the article.

## Supplementary Materials

The following are available online at https://www.mdpi.com/article/10.3390/polym14010053/s1.
