# Supplementary material for: Phase Change Energy Storage Elastic Fiber: A Simple Route to Personal Thermal Management
Source: Polymers (Basel). 2021 Dec 24;14(1):53. doi: 10.3390/polym14010053 (PMC8747497; doi:10.3390/polym14010053)
Supplement: Supplementary file 1 [file polymers-14-00053-s001.zip › polymers-1474746-supplementary.pdf]

Supplementary materials

Table S1. The formulation of the elastic TPU scaffolds.

Scaffolds	TPU/g	DMF/ml
TPU-0.20	5	25
TPU-0.28	7	25
TPU-0.40	10	25

Table S2. Phase change behavior of fibers with different spinning solution concentration and different PCMs.

Sample	T_{mo}(℃)	T_{mp}(℃)	ΔH_m(J/g)	T_{co}(℃)	T_{cp}(℃)	ΔH_c(J/g)	*E* (%)
OCC	26.4	31.4	226	25.4	21.6	230.1	-
OCC/TPU-0.2	25.8	37.2	176.1	24.1	17.1	174.6	76.9
OCC/TPU-0.28	26.4	35.0	154.2	25.0	17.9	157.8	68.4
OCC/ TPU-0.4	26.3	33.7	127.7	24.8	18.7	128.8	56.2
HEO	45.9	51.5	263.9	47.1	43.6	262	-
HEO/TPU-0.2	45.2	52.7	208.1	47.3	40.6	210.7	79.6
HEO/TPU-0.28	45.4	56.7	177.8	46.8	37.4	185.1	69.0
HEO/ TPU-0.4	44.2	55.5	157.2	47.0	39.4	160.7	60.4
SA	66.2	71.2	206.9	66	61.7	210.6	-
SA/TPU-0.2	65.3	73.3	174	64.5	59.2	177.5	84.2
SA/TPU-0.28	65.8	73.1	157.1	64.7	59.1	161.4	76.3
SA/TPU-0.4	64.9	73.4	148.3	63.8	57.9	152.5	72.0

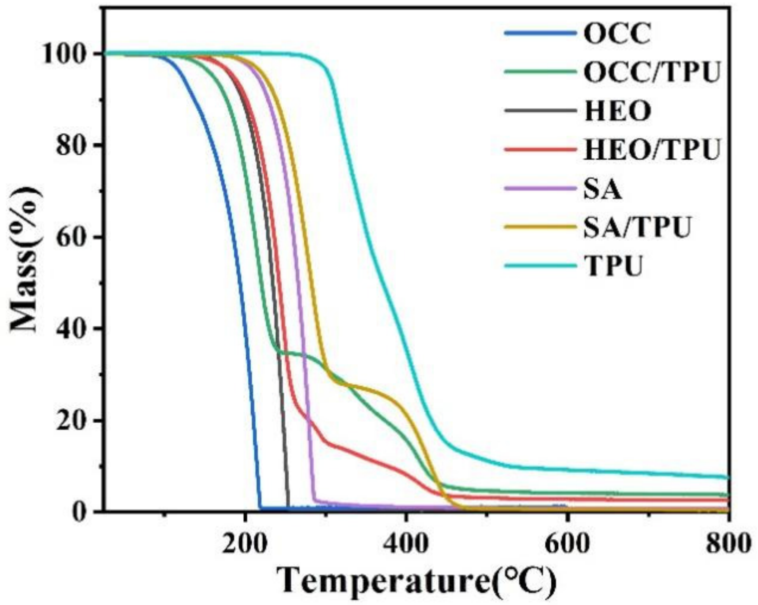

Figure S1. TGA curves of TPU-0.28, Y/TPU-0.28 fibers.

Table S3. Degradation data of weight loss of PCMs, TPU-0.28 and Y/TPU-0.28 fibers

Sample	Initial weight loss temperature (°C)	Second weight loss temperature (°C)	Residual mass(%)
OCC	126.1	-	0.85
OCC/TPU-0.28	183.4	297.1	3.85
HEO	210.7	-	0.61
HEO/TPU-0.28	216.6	299.2	2.57
SA	247.1	-	0.65
SA/TPU-0.28	249.5	306.9	0.96
TPU	301.6	-	6.64

Table S4. Mechanical properties of different fibers

Sample	Breaking strength (MPa)	Elongation at break (%)	Initial modulus (KPa)
TPU-0.2	2.46±0.30	433.9±23.4	16.12±1.51
TPU-0.28	3.30±0.18	439.3±21.5	17.41±1.63
TPU-0.4	5.50±0.25	522.1±25.1	20.43±2.45
OCC/TPU-0.2 (Frozen)	2.12±0.17	364.3±19.2	25.19±2.89
OCC/TPU-0.28 (Frozen)	2.64±0.19	410.0±21.4	30.98±4.13
OCC/TPU-0.4 (Frozen)	4.18±0.23	478.3±24.7	36.57±3.79
OCC/TPU-0.2 (Molten)	2.37±0.27	394.7±18.9	16.81±2.14
OCC/ TPU-0.28(Molten)	2.78±0.22	425.1±29.4	18.56±1.43
OCC/TPU-0.4(Molten)	4.54±0.14	496.5±23.8	22.32±1.24
HEO/TPU-0.2 (Frozen)	0.99±0.12	161.6±15.1	50.13±4.19
HEO/TPU-0.28(Frozen)	1.67±0.12	354.8±19.9	70.52±5.87
HEO/TPU-0.4(Frozen)	2.65±0.21	458.7±22.7	81.28±4.54
SA/ TPU-0.2 (Frozen)	1.34±0.13	253.4±14.8	61.29±4.63
SA/ TPU-0.2 8(Frozen)	2.27±0.11	374.2±21.1	77.93±5.16
SA/TPU-0.4 (Frozen)	3.36±0.21	498.0±14.4	85.74±5.72
